# Acetyl-11-Keto-*β*-Boswellic Acid (AKBA) Prevents Lipopolysaccharide-Induced Inflammation and Cytotoxicity on H9C2 Cells

**DOI:** 10.1155/2022/2620710

**Published:** 2022-03-30

**Authors:** Danial Taherzadeh, Vafa Baradaran Rahimi, Hamed Amiri, Sajjad Ehtiati, Roghayeh Yahyazadeh, Seyed Isaac Hashemy, Vahid Reza Askari

**Affiliations:** ^1^Department of Clinical Biochemistry, Faculty of Medicine, Mashhad University of Medical Sciences, Mashhad, Iran; ^2^Surgical Oncology Research Center, Mashhad University of Medical Sciences, Mashhad, Iran; ^3^Department of Cardiovascular Diseases, Faculty of Medicine, Mashhad University of Medical Sciences, Mashhad, Iran; ^4^Applied Biomedical Research Center, Mashhad University of Medical Sciences, Mashhad, Iran; ^5^Department of Pharmaceutical Sciences in Persian Medicine, School of Persian and Complementary Medicine, Mashhad University of Medical Sciences, Mashhad, Iran; ^6^Department of Persian Medicine, School of Persian and Complementary Medicine, Mashhad University of Medical Sciences, Mashhad, Iran

## Abstract

Acetyl-11-keto-beta-boswellic acid (AKBA), the major component of *Boswellia serrata*, exhibits anti-inflammatory activities. This in vitro study investigated the protective effects of AKBA against lipopolysaccharide (LPS)-induced cardiac dysfunction. In this study, the H9C2 cardiomyocytes were pretreated with AKBA (2.5, 5, and 10 *μ*M for 24 h), and then cotreated with LPS for another 24 h. The MTT assay, ELISA test kits, and quantitative real-time PCR analysis assessed the cell viability, levels of proinflammatory factors (IL-*β*, IL-6, TNF- *α*, and PGE2), and the gene expression of IL-*β*, IL-6, TNF- *α*, iNOS, and COX-2, respectively. The nitric oxide (NO) and thiol levels were also measured using a biochemical assay. The results indicated that LPS exposure markedly reduced cell viability and total thiol content, but increased the inflammatory cytokines, NO metabolites, and gene expression of proinflammatory mediators in H9C2 cells. AKBA pretreatment significantly altered the mentioned factors induced by LPS. Our results demonstrated that AKBA might be a promising therapeutic agent for treating sepsis-related cardiac dysfunction in the future.

## 1. Introduction

Sepsis is a lethal condition caused by an overreaction of the immune system to infection that can lead to tissue damage, multiple organ failure, and even death [1–4]. Myocardial dysfunction is one of the prominent sepsis features that has proved to be associated with a high mortality risk for septic patients [5, 6]. Many studies have reported the crucial role of proinflammatory cytokines in myocardial oxidative damage that commonly occurs in the course of sepsis [7]. Throughout these severe inflammatory conditions, the elevating production of multiple proinflammatory mediators, such as interleukin-1beta (IL-1*β*), IL-6, tumour necrosis factor-*α* (TNF-*α*), and nitric oxide (NO), eventually lead to cardiac depression, oedema, and necrosis in the myocardial cells [2–4, 8]. According to previous studies, the systemic inflammatory response is stimulated by damage-associated molecular patterns (DAMPs) such as lipopolysaccharide (LPS) as a bacterial endotoxin, which can induce inflammatory signalling pathways through toll-like receptor-4 (TLR-4) in cardiomyocytes [3, 9, 10]. The LPS/TLR-4 complex formation can activate further inflammatory signalling cascades associated with several transcription factors, particularly the nuclear factor-kappa B (NF-*κ*B) pathway, which stands as the primary signalling cascade in initiating the process of intracellular inflammation [3, 10, 11]. Considering the activation of inflammation by NF-*κ*B, the intensified proinflammatory cytokines can accumulate intracellular oxygen free radicals (ROS) and automatically impair the structure and function of cardiomyocytes [7, 10, 12–14]. The available pharmacological approaches, such as using nonsteroidal anti-inflammatory drugs (NSAIDs), are minimal due to their controversial and various adverse effects [7, 10, 13–15]. It is consequently necessary to consider efficient therapeutic interventions to prevent sepsis-induced cardiomyopathy. There is growing evidence on natural compounds, especially plants' secondary metabolites, are capable of representing optimal therapeutic effects in alleviating LPS-induced cardiotoxicity [6, 16].

Acetyl-11-keto-*β*-boswellic acid (AKBA), as a plant-derived-bioactive pentacyclic triterpene, has been isolated from *Boswellia serrata* (BS) [4, 10, 17]. The remarkable biological features of AKBA have been repeatedly indicated, including antioxidant [18], antitumour [19], antimicrobial activity [20], anti-inflammatory [4, 10, 13, 14], neuroprotective [2, 14], and other beneficial qualities [13, 14, 21]. Recently, the cardioprotective activities of pentacyclic triterpenoid compounds have been paid the attention of many researchers. In one study, two natural compounds with a similar chemical structure (boswellic acid and oleanolic acid) have been investigated on high glucose-induced toxicity in the H9C2 cardiomyocyte cell line. This study showed that these compounds attenuate apoptosis by reducing the activity of NF-*κ*B, lowering ROS production, and enhancing the glutathione redox cycle [22]. Further studies also reported the protective impacts of AKBA against the consequences of myocardial ischemia-reperfusion (I/R) injury in a rat model by modulating the oxidative-inflammatory cascades [23]. Based on the abovementioned data, this work aimed to investigate the effects of AKBA on lipopolysaccharide (LPS)-induced cell injury in H9C2 cells.

## 2. Methods and Materials

### 2.1. Chemicals and Reagents

AKBA (Calbiochem), DMEM culture media, fetal bovine serum (FBS), penicillin plus streptomycin (pen/strep), dimethyl sulfoxide (DMSO), LPS (*Escherichia coli* O55 : B5 purified by phenol extraction, L2880 SIGMA), and other chemicals used were of cell culture and analytical grade from Sigma-Aldrich (St. Louis, MO, USA). A proliferation assay kit (MTT) was provided from Roche Diagnostic (Mannheim, Germany). ELISA kits (PGE2, IL-6, IL-1*β*, and TNF-*α*) were supplied from IBL International (USA).

### 2.2. Cell Viability Assay

In this study, we first evaluated the viability of H9C2 cells in the presence of AKBA (2.5, 5, and 10 *μ*M) to better understand the working solution selected based on nontoxic concentration. In brief, the cells were treated with different concentrations of AKBA (2.5, 5, and 10 *μ*M) for 48 h, and then the cell viability was evaluated employing the MTT assay. Once the nontoxicity of the applied concentrations towards cells was ascertained, the protective effects of AKBA were assessed. In the next step, we evaluated the effects of the concentrations of AKBA (2.5, 5, and 10 *μ*M) on LPS-induced (10 *μ*g/ml) by culturing 7 × 103 H9C2 cells in a 96-well plate. In review, the cells were pretreated with different concentrations of AKBA (2.5, 5, and 10 *μ*M) for 24 h, and then were cocultured with LPS (10 *μ*g/ml) for another 24 h. In the end, the cell viability was measured by applying an MTT assay. For the MTT assay, briefly, 10 *μ*L of MTT solution with a final concentration of 5 mg/ml was appended to each well to be incubated for 3 h. After discarding the medium culture (RPMI-1640), 100 *μ*L of DMSO was used to dissolve the formed formazan crystals. The absorption of the 96-well plate was recorded by an ELISA reader (Awareness Inc., USA) at 570 nm and 620 nm [7].

### 2.3. Experimental Procedure and Grouping

The protective effects of AKBA against LPS-induced cardiomyocyte toxicity were evaluated as a model of septic shock. Initially, the cells were pretreated with AKBA (2.5, 5, and 10 *μ*M) for 24 h, and then were coexposed with LPS (10 *μ*g/ml) for another 24 h. After that, we assessed the changes in both gene expression (in the cell lysate, using real-time PCR) and protein (in the supernatant, using ELISA) levels of proinflammatory biomarkers, including TNF-*α*, IL-1*β*, IL-6, PGE2, iNOS, COX-2, and nitric oxide metabolites (NO). Total thiol content was also assessed as an antioxidant marker in the lysate. Experimental groups were as follows:

  Group 1: control group, H9C2 cells received a complete media culture and the solvent of AKBA with neither AKBA nor LPS for 48 h
  Group 2: LPS group, H9C2 cells received a complete media culture and the solvent of AKBA and LPS (10 *μ*g/ml) for 48 h
  Groups 3, 4, and 5: AKBA treated groups, H9C2 cells received a complete media culture and AKBA (2.5, 5, and 10 *μ*M) for 24 h, and then coincubated with LPS (10 *μ*g/ml) for another 24 h

AKBA was dissolved in DMSO, which was serially diluted with a complete medium that contained the final DMSO concentration at a lower percentage than 0.1% v/v throughout all of the experiments. We selected the concentrations of AKBA according to the preliminary results of the cell viability by the MTT assay, and a similar study evaluated the antioxidative effects of AKBA (2.5–10 *μ*M) [16].

### 2.4. Measuring Total Protein Levels

The Bradford protein assay was carried out to quantify the total protein concentration in a sample using the Coomassie Brilliant Blue G-250 dye [24–27]. First, the dye (10 mg) was dissolved in 50 ml of ethanol (96%), and then phosphoric acid (85%) (10 ml) was added, and the volume of the solution reached 100 ml. Thereafter, bovine serum albumin (BSA, 4 mg/ml) solution was prepared as a standard curve. Then, after sample pouring (20 *μ*l), a Bradford reagent (200 *μ*l) was added to the 96-well microplate. Finally, after 5 minutes, the absorption was read out at 595 nm with a microplate reader [24–27].

### 2.5. Evaluation of the Protein Levels of Inflammatory Biomarkers

The ELISA assay evaluated the IL-1*β*, IL-6, TNF-*α*, and PGE2 levels as inflammatory mediators, which were carried out in accordance with the manufacturers' protocol, IBL company [6, 7, 24, 26, 28], USA. In summary, 1.5 × 10^6^ cells were cultured in a 6-well plate overnight, and they were treated according to the experimental grouping section. The supernatant of cells was then used for the required measurements, and the lysates were collected for gene expression studies.

### 2.6. Gene Expression Assessment

A real-time PCR technique was performed through the SYBR Green procedure's employment to assess the possible impacts of different AKBA concentrations on the levels of TNF-*α*, IL-1*β*, IL-6, and COX-2 as well as iNOS related mRNA. Rotor-Gene 6000 was involved as a real-time reaction detection system, and GAPDH was considered a reference gene [7, 24, 26]. We procured the required real-time PCR primers in a similar design to those mentioned in previous studies [29]; the primer specificity was blasted and confirmed by applying NCBI Primer-BLAST. The primer sequences are detailed in Table 1. The real-time PCR reaction contained 5 *μ*L of amplicon master mix, 0.4 *μ*L of each primer (1 *μ*M), 0.2 *μ*L of DEPC water, and 50 ng of cDNA. Meanwhile, the prepared conditions for PCR were set at 95°C for 3 min and then followed by 45 cycles of 95°C for 20 sec, annealing temperature (55–65°C) for 5 sec, and 72°C for 10 sec. As the last step, we examined the values of gene expression levels by using the ∆Ct method and reported the fold-change values as 2^−(∆∆Ct)^ to the control group.

### 2.7. Evaluation NO Metabolites and Thiol Content Level

The nitrite oxide metabolite levels were measured based on the measurement of nitrite (NO^−2^) as the stable and the final NO product by the Griess method described elsewhere [30]. The NO levels were assessed in the supernatant at 540 nm by using the standard curve of different nitrite concentrations [6, 7, 24, 26–28].

The total thiol content was evaluated through a colourimetric method, which was set according to the reaction of total thiol content with Ellman reagent (5,5′-dithiobis (2-nitrobenzoic acid) (Sigma-Aldrich) [31]. This particular reaction results in the formation of yellow-coloured TNB (5-thio-2-nitrobenzoic acid) that can be quantified at 412 nm [6, 7, 24, 26–28].

### 2.8. Statistical Analysis

We displayed the obtained results as mean ± SEM. The gathered data were analysed by GraphPad Prism 6 (GraphPad Software, San Diego, CA, USA) software. Besides, the one-way analysis of variance (ANOVA) test was carried out with Tukey-Kramer's post hoc multiple comparisons test according to the variance's homogeneity. By statistics, probability (*P*) values of less than 0.001, 0.01, and 0.05 were considered significant differences in all of the performed calculations.

## 3. Results

### 3.1. AKBA Alleviates LPS-Induced Cytotoxicity in H9C2 Cells

**Figure 1:**
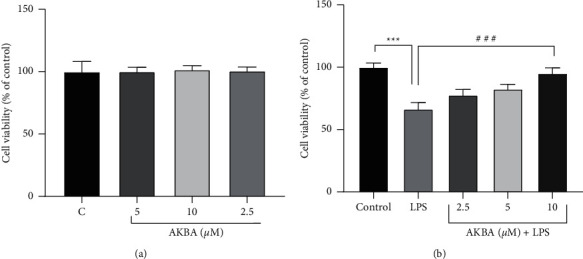
H9C2 cell viability in the presence of AKBA alone and with LPS. Effect of AKBA (2.5–10 uM) on cell viability (a). The survival of the H9C2 cell line was evaluated when treated with LPS (10 ug/ml) and 24 h of pretreatment with AKBA then exposure with LPS (b). n = six per group. Error bars indicate standard error mean (SEM) ^*∗∗∗*^*P* < 0.001 vs. control and ^###^*P* < 0.001 vs. LPS.

As shown in Figure 1(a), in comparison to the control group, there were no significant changes in the level of cell viability of H9C2 cells incubated with various concentrations of AKBA (2.5, 5, and 10 *μ*M) for 48 h. Incubation of the cells with LPS (10 *μ*g/mL) for 48 h led to a significant reduction in the level of cell viability compared to the control group (*P* < 0.001; Figure 1(b)). However, treatment with AKBA (5 and 10 *μ*M) notably increased the level of cell viability in the presence of LPS stimulation (*P* < 0.05 and 0.001, respectively; Figure 1(b)).

### 3.2. AKBA Inhibits LPS-Induced Inflammatory Cytokines Production in H9C2 Cells

**Figure 2:**
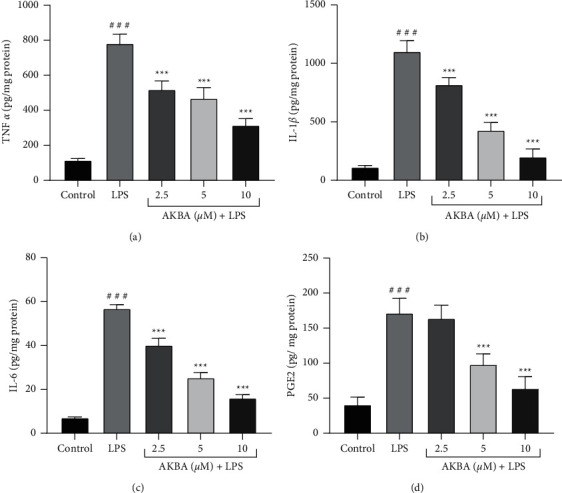
Effect of AKBA and LPS on the level of TNF-*α* (a), IL-1*β* (b), IL-6 (c), and PGE2 (d). The cells were pretreated with AKBA for 24 h, then incubated with LPS for another 24 h, and levels of TNF-*α*, IL-1*β*, IL-6, and PGE2 were evaluated by an ELISA assay. The data are the mean ± SEM (*n* = 6 per group). ^###^*P* < 0.001 vs. control and ^*∗∗∗*^*P* < 0.001 vs. LPS.

To demonstrate the anti-inflammatory effects of AKBA, we assessed the production levels of proinflammatory cytokines, including TNF-*α*, IL-6, IL-1*β*, and PGE2, which contributed to LPS-induced cardiomyopathy. As illustrated in Figures 2(a)–2(d), the production levels of TNF-*α*, IL-6, IL-1*β*, and PGE2 were significantly elevated in H9C2 cells following the LPS (10 *μ*g/mL) stimulation in comparison to the control group (*P* < 0.001 for all cases). However, pretreatment of the cells with AKBA (2.5, 5, and 10 *μ*M) dramatically decreased the production of TNF-*α* (*P* < 0.001 for all cases; Figure 2(a)), IL-1*β* (*P* < 0.001 for all cases; Figure 2(b)), and IL-6 (*P* < 0.001 for all cases; Figure 2(c)) in a concentration-dependent manner, compared to the LPS group. Although AKBA exerted reducing effects on the level of PGE2, this effect was statistically significant only at two higher concentrations of AKBA (5 and 10 *μ*M) in comparison to the LPS group (*P* < 0.001 for both cases; Figure 2(d)).

### 3.3. AKBA Attenuates the Gene Expression Levels of Inflammatory Cytokines in H9C2 Cells

**Figure 3:**
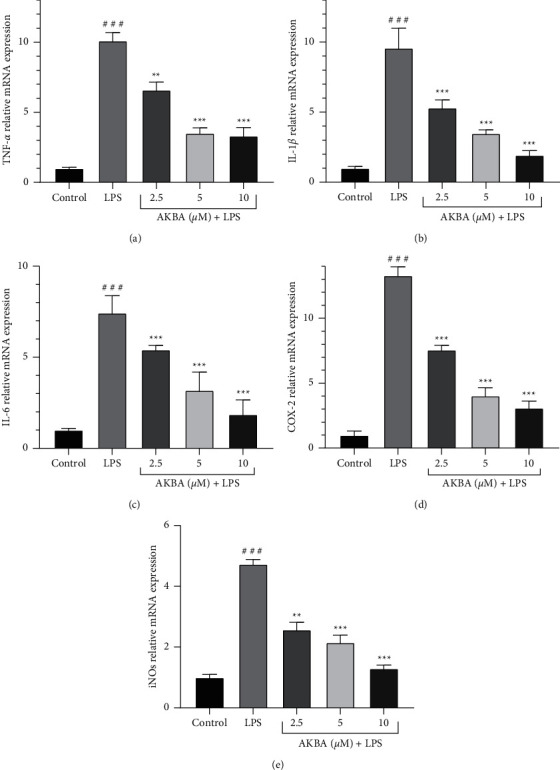
Effect of AKBA and LPS on the mRNA Level of TNF-*α* (a), IL-1*β* (b), IL-6 (c), Cox2 (d), and iNos (e). The cells were pretreated with AKBA for 24 h then incubated with LPS for another 24 h and gene expression of TNF-*α*, IL-1*β*, IL-6, Cox2, and iNos was evaluated by real time-PCR. The data are mean ± SEM (*n* = 6 per group). ^###^*P* < 0.001 vs. control, ^*∗∗*^*P* < 0.001 and ^*∗∗∗*^*P* < 0.001 vs. LPS.

Our study evaluated the capability of AKBA in suppressing the transcription of proinflammatory mediators in our study using the real-time PCR (qPCR) technique. According to Figures 3(a)–3(e), treatment of H9C2 cells with LPS (10 *μ*g/mL) caused a significant enhancement in proinflammatory genes' expression levels (IL-1*β*, TNF-*α*, IL-6, iNOS, and COX-2). We revealed that three nontoxic concentrations of AKBA (2.5, 5, and 10 *μ*M) exhibited notable suppressive impacts on the expression levels of proinflammatory mediators (IL-1*β*, TNF-*α*, IL-6, iNOS, and COX-2), in a concentration-dependent manner, compared to the LPS group (*P* < 0.001–0.01 for all cases; Figures 3(a)–3(e)).

### 3.4. AKBA Decreased Nitric Oxide Level and Increased Thiol Level

**Figure 4:**
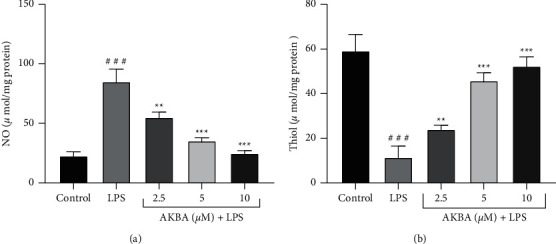
Cellular redox status including thiol state and NO state after treatment with AKBA and LPS. The cells were treated (24 hours) with various concentrations AKBA (2.5–10 *μ*M) and then incubated with LPS (10 ug/ml) for 24 hours. The data are mean ± SEM (*n* = 6). ^###^*P* < 0.001 vs. control, ^*∗∗*^*P* < 0.001 and ^*∗∗∗*^*P* < 0.001 vs. LPS.

We investigated the antioxidant effect of AKBA in LPS-stimulated H9C2 cells by detecting the levels of nitric oxide (NO) and thiol [32] in the culture medium. According to the obtained results, LPS at a concentration of 10 *μ*g/mL led to a significant enhancement in the level of NO (*P* < 0.001; Figure 4(a)) and a significant reduction in the level of total thiol content (*P* < 0.001; Figure 4(b)), compared to the control group.

In contrast, pretreatment with AKBA (2.5, 5, and 10 *μ*M) caused a significant inhibition of NO production compared to the LPS-treated group (*P* < 0.001–0.01 for all cases; Figure 4(a)). Moreover, preincubation of the cells with AKBA (2.5, 5, and 10 *μ*M) resulted in a marked increase in the levels of total thiol content in a concentration-dependent manner compared to the LPS group (*P* < 0.001–0.01 for all cases; Figure 4(b)).

## 4. Discussion

To the best of our knowledge, the present work is the first study to investigate the potential protective effects of AKBA against LPS-induced cardiomyopathy. Here, we used an in vitro H9C2 model of myocardial injury to explore the protective mechanism of AKBA on LPS-induced cardiac dysfunction [33]. Our study results showed that LPS increased the levels of proinflammatory mediators (IL-1*β*, TNF-*α*, IL-6, and PGE2) and nitric oxide (NO), whereas it reduced the cell viability of H9C2 cells as well as the level of total thiol content. However, pretreatment with various concentrations of AKBA (2.5, 5, and 10 *μ*M) could dramatically enhance the cell viability and the content of total thiol content. Furthermore, AKBA significantly suppressed the expression of proinflammatory factors and NO production, indicating that AKBA might be effective against LPS-induced inflammatory responses. We also found that the gene expression of COX-2 and iNOS was down-regulated by AKBA pretreatment.

It is well established that LPS, as an exogenous ligand of TLR4, induces inflammatory processes involved in severe cardiac injury [34]. LPS-mediated activation of initiates NF-*κ*B dependent signalling pathways, resulting in the overproduction of various proinflammatory mediators, such as COX-2, iNOS, IL-1*β*, IL-6, and TNF-*α* [6, 24, 26–28, 35, 36]. Excessive production of proinflammatory cytokines plays a vital role in developing many inflammatory disorders, including sepsis-induced myocardial dysfunction [37]. Meanwhile, several studies have investigated the role of TNF-*α* in the pathogenesis of LPS -induced acute cardiac injury and have shown that high concentrations of TNF-*α* promoted the expression of specific cytokines and mediators involved in LPS-induced septic cardiomyopathy [6, 24, 26–28, 38, 39]. IL-6 is another important proinflammatory factor that can directly cause myocardial damage by stimulating nitric oxide synthase activity and NO production [40]. Besides, several published studies have reported that cytokines such as IL-1*β* may act as cardiodepressant inflammatory mediators during sepsis [6, 24, 26–28, 41]. Hence, inhibiting the production of inflammatory cytokines can be a critical molecular target for novel anti-inflammatory therapeutic approaches. In the present study, LPS notably stimulated the release of proinflammatory cytokines from H9C2 cardiomyocytes, consistent with previous reports [42]. The anti-inflammatory activity of AKBA has been investigated using several models of inflammation. Previous studies have reported that AKBA inhibits the generation of proinflammatory cytokines through down-regulation of the NF-*κ*B pathway [43]. For example, Chao Wei et al. evaluated the neuroprotective function of AKBA in a mouse model of Alzheimer's disease. Their findings indicated that AKBA had a strong anti-inflammatory effect on APPswe/PS1dE9 mice by reducing inflammatory molecules' production through the inhibition of the NF-*κ*B signalling pathway [44]. It has also been reported that the potential cardiac-protective of AKBA is likely associated with the activation of Nrf-2 related signalling cascades [23]. In another study, AKBA was shown to effectively alleviate oxygen-glucose deprivation (OGD)-induced neuroinflammation via the increased expression of Nrf2 [45].

Meanwhile, we explored the possible cardiac-protective effect of AKBA against LPS-induced inflammatory cytokine production in H9C2 cells. The results showed that AKBA pretreatment dramatically suppressed the protein and gene expression of IL-1*β*, IL-6, and TNF-*α* in LPS-exposed H9C2 cells in a concentration-dependent manner. Our results revealed that the protective effects of AKBA against LPS-induced cardiac injury were associated with inflammatory cytokine production inhibition. The obtained results were in accordance with a previous study in which AKBA markedly reduced TNF-*α* production in the target tissue against the LPS-induced neuroinflammatory model [13]. These findings suggest that the potent anti-inflammatory properties of AKBA may be related to its inhibitory activity on the expression of inflammatory factors.

COX-2 is an inducible isoform from cyclooxygenase that catalyses the formation of prostaglandin E2 (PEG_2_), which is involved in many processes leading to the inflammatory response [6, 24, 26–28, 46]. Many studies have shown that COX-2 enzymatic activity is significantly induced by proinflammatory stimuli such as TNF- *α* and LPS and high NO concentrations during various inflammatory conditions [47–49]. Thus, the level of COX-2 expression seems to play a pivotal role in multiple pathophysiological mechanisms, especially inflammation-related diseases [49, 50]. Likewise, several studies have proved that many natural products derived from medicinal plants with significant anti-inflammatory effects act as selective inhibitors of COX-2 activity [6, 24, 26–28, 49, 51]. In this research, we found that AKBA could efficiently suppress the mRNA expression of COX-2 in H9C2 cardiomyocytes activated with LPS. This outcome supports the hypothesis that the cardiac-protective effect of AKBA may also be attributed to the direct suppression of COX-2 gene expression. To further investigate the anti-inflammatory potential of AKBA, we measured the expression level of PEG2 in AKBA + LPS-treated H9C2 cells. Results indicated that pretreatment with AKBA meaningfully decreased the PGE2 level in LPS-activated H9C2 cells. As a result, inhibiting these inflammatory mediators' production can be an essential indicator for our anti-inflammatory agents.

Inducible nitric oxide synthase (iNOS) is commonly expressed in response to proinflammatory stimuli such as LPS and specific chemokines/cytokines in a wide range of cells. It has been found that the overproduction of NO by iNOS activity plays a crucial role in the pathophysiology of septic cardiomyopathy [52]. High NO levels lead to the induction of cellular oxidative damage via increasing the production of reactive nitrogen species (RNS) such as peroxynitrite [53]. Many previous studies have revealed that natural phytochemicals can show therapeutic effects against LPS-evoked inflammatory injury via reducing the iNOS expression and NO content [54]. In the current study, we also found a significant suppressive effect of AKBA on the expression of iNOS and the production of NO.

Similarly, antioxidant effects of AKBA have been previously reported. Manoj Kumar et al. have demonstrated that AKBA inhibits benzo (a) pyrene-induced liver toxicity by reducing NO generation [55]. Besides, Yu et al. investigated the nitric oxide inhibitory activity of bioactive compounds isolated from the resin of *Boswellia carteri*. Interestingly, their experiments showed that AKBA was one of the most potent antioxidant compounds in *Boswellia carteri* that could inhibit NO production in LPS-stimulated RAW264. 7 cells [56]. These findings provide novel evidence that AKBA may attenuate inflammatory responses by suppressing iNOS-mediated NO production.

Additionally, the amount of glutathione is another parameter used in our study to evaluate the antioxidant activity of AKBA. As a nonenzymatic antioxidant compound, total thiol content has a crucial role in protecting cells from oxidative stress and maintaining cellular redox homeostasis [57]. It has been proven that a decreased level of reduced total thiol content leads to activation of the NF-*κ*B pathway and enhances the expression of proinflammatory cytokines during inflammation-related disorders [58]. Consequently, the increased level of total thiol content can be part of the antioxidant defence mechanism to inhibit sepsis-induced cardiomyopathy [59]. On the other hand, natural metabolites have been shown to protect against oxidative damage in many inflammatory conditions by enhancing intracellular total thiol content [6, 24, 26–28, 49, 60]. The results of a previous study revealed that AKBA possessed antioxidant effects through elevating the reduced total thiol content levels in an experimental model of colitis [61]. Consistent with these findings, we also observed a remarkable increase of reduced total thiol content in LPS-exposed H9C2 cardiomyocytes after pretreating with AKBA. Based on this result, it can be inferred that the beneficial effects of AKBA on LPS-induced inflammation in H9C2 cells may be due to its potent antioxidant potential.

Overall, our findings demonstrate the potent anti-inflammatory and antioxidant properties of AKBA, suggesting that it can be used as an effective therapeutic agent to lessen the inflammatory injuries caused by LPS in cardiomyocytes. However, additional in vivo experiments are required to further support the therapeutic potential of AKBA in sepsis-related myocardial dysfunction.

## Figures and Tables

**Table 1:** List of primers sequence (from 5′ to 3′) [7, 29].

Gene name	5′-3′ primer	Sequence Accession Number
*tnf-α*	FW CACCTCTCAAGCAGAGCACAG RW GGGTTCCATGGTGAAGTCAAC	M98820
*cox-2*	FW AAATGGGCTCCCTCTCATCAGTTC RW TCTGCTTGGTGGTTTGCTACGAC	X66539
*il-1β*	FW TGTATGCTACCATCTGGCTTCGG RW GTTTGGAACAGTCGCTCGTCATC	S67722
*il-6*	FW CATTGGAAGTGAAGCGTTTCG RW CAGCTGGGCTGTACAAACCTT	L12562
*inos*	FW TCCTACCCCAACTTCCAATGCTC RW TTGGATGGTCTTGGTCCTTAGCC	E02522
*gapdh*	FW GTATTGGGCGCCTGGTCACC RW CGCTCCTGGAAGATGGTGATGG	AB017801

FW, forward primer; RW, reverse primer; GAPDH, glyceraldehyde-3-phosphate dehydrogenase.

## Data Availability

The data used to support the findings of this study are available from the corresponding author upon reasonable request.
